# Market integration and soil-transmitted helminth infection among the Shuar of Amazonian Ecuador

**DOI:** 10.1371/journal.pone.0236924

**Published:** 2020-07-31

**Authors:** Theresa E. Gildner, Tara J. Cepon-Robins, Melissa A. Liebert, Samuel S. Urlacher, Joshua M. Schrock, Christopher J. Harrington, Felicia C. Madimenos, J. Josh Snodgrass, Lawrence S. Sugiyama

**Affiliations:** 1 Department of Anthropology, Dartmouth College, Hanover, New Hampshire, United States of America; 2 Department of Anthropology, University of Colorado, Colorado Springs, Colorado, United States of America; 3 Department of Anthropology, Northern Arizona University, Flagstaff, Arizona, United States of America; 4 Department of Anthropology, Baylor University, Waco, Texas, United States of America; 5 Department of Anthropology, University of Oregon, Eugene, Oregon, United States of America; 6 Department of Anthropology, Queens College (CUNY), Flushing, New York, United States of America; 7 Center for Global Health, University of Oregon, Eugene, Oregon, United States of America; Dokkyo Medical University, JAPAN

## Abstract

**Background:**

Soil-transmitted helminth (STH) infections have many negative health outcomes (e.g., diarrhea, nutritional deficiencies) that can also exacerbate poverty. These infections are generally highest among low-income populations, many of which are also undergoing market integration (MI; increased participation in a market-based economy). Yet the direct impact of MI-related social and environmental changes on STH infection patterns is poorly understood, making it unclear which lifestyle factors should be targeted to better control disease spread. This cross-sectional study examines if household infrastructure associated with greater MI is associated with lower STH burdens among Indigenous Ecuadorian Shuar.

**Methods:**

Kato-Katz fecal smears were used to determine STH infection status and intensity (n = 620 participants; 308 females, 312 males, aged 6 months—86 years); *Ascaris lumbricoides* (ascarid) and *Trichuris trichiura* (whipworm) were the primary infection types detected. Structured interviews assessing lifestyle patterns (e.g., measures of household infrastructure) measured participant MI. Multilevel regression analyses and zero-inflated negative binomial regression models tested associations between MI measures and STH infection status or intensity, controlling for individual and community characteristics.

**Results:**

Participants residing in more market-integrated households exhibited lower infection rates and intensities than those in less market integrated households. Parasite infection status and *T*. *trichiura* infection intensity were lower among participants living in houses with wood floors than those with dirt floors, while individuals using well or piped water from a spring exhibited lower *A*. *lumbricoides* infection intensities compared to those using river or stream water. Unexpectedly, latrine type was not significantly related to STH infection status or intensity. These results suggest that sources of exposure differ between the two helminth species.

**Conclusions:**

This study documents associations between household measures and STH infection among an Indigenous population undergoing rapid MI. These findings can help healthcare programs better target interventions and reduce STH exposure among at-risk populations.

## Introduction

Populations transitioning from traditional subsistence practices to more industrialized lifestyles generally experience rapid economic, social, and nutritional changes including agricultural intensification, increased consumption of market foods, and the adoption of new languages and values [1–5]. These changes are collectively referred to as Market Integration (MI; the degree to which people consume from and produce for a market economy) and may have profound implications for population health and well-being [6–11]. For example, MI-induced cultural change affects the way a group understands and interacts with their environment (e.g., altering hygiene practices and the way buildings are constructed), thereby altering disease pathways and risk of infection [2,12,13]. Lifestyle factors associated with MI consequently have the potential to impact disease risk drastically, in both positive and negative ways.

To date, most research examining the relationship between MI and human health has focused on chronic diseases, including obesity, type 2 diabetes, hypertension, and cardiovascular disease [14–20]. While these health changes are important, populations experiencing rapid MI are often characterized by both elevated chronic and infectious disease burdens, resulting in a “double burden” of disease [21–24]. The design of more effective intervention programs can therefore benefit from research identifying specific factors associated with MI that strongly influence infectious disease risk. To ensure the greatest impact, these research efforts must focus attention on infectious diseases commonly found in lower-income populations undergoing MI.

Different aspects of MI may either decrease or increase levels of pathogen exposure [13]. Some changes linked with MI (e.g., improved sanitation systems, clean water, houses with paved floors and window screens, access to commercialized hygiene products, and improved medical care access) are expected to decrease infection risk from a range of pathogens (e.g., cholera, Chagas disease, Dracunculiasis, ascariasis) [2,5,25–29]. Conversely, other changes (e.g., higher population densities, increased travel, greater reliance on domesticated animals, deforestation/increased standing water reservoirs) may instead favor the transmission of certain pathogens (e.g., malaria, viral infections, lymphatic filariasis, trichuriasis) [2,27,28,30–33]. Market integration may therefore have mixed effects on infectious disease patterns.

Parasitic infections, in particular, are often endemic in areas around the world characterized by substantial poverty; this pattern includes high infection rates of soil-transmitted helminths (STHs; intestinal parasitic worms) [34,35]. Notably, STHs infect more than a quarter of the global population, with millions of individuals infected with a few key species: ascarid (*Ascaris lumbricoides*, ~800 million cases globally), whipworm (*Trichuris trichiura*, ~400 million cases globally), and hookworm (*Ancylostoma duodenale* and *Necator americanus*, ~400 million cases globally) [35–39]. Soil-transmitted helminth infections are spread by fecal-oral contamination (i.e., through water, food, or soil that has been contaminated with fecal matter from infected humans or other animals). While these conditions do not typically result in death, heavier infections can cause negative health outcomes including nutritional deficiencies, stunted growth, developmental delays, diarrhea, intestinal blockages, and organ failure [40–45].

Furthermore, STH infection has been linked with suppressed immune function, leading to an elevated risk of infection with other pathogens and reduced vaccine efficacy [46]. Morbidity associated with STH infection also promotes poverty through several pathways, including lower educational attainment, decreased work productivity, and reduced future wage-earning capacity, trapping affected populations in a cycle of disease and poverty [47,48]. It is therefore unsurprising that this category of infectious disease ranks near the top of global health threats [49]. Yet STHs remain classified as “neglected tropical diseases” because relatively little attention has been devoted to them [47]. The lack of medical investment in STHs is due, in part, to the fact that endemic diseases suffered by people living in poverty often remain invisible to policymakers and those who distribute the majority of global medical resources [50]. Because of this lack of investment, it remains unclear which lifestyle factors should be targeted to decrease STH transmission rates most effectively.

To address this critical global health issue, the present study provides data on the links between soil-transmitted helminthiasis and lifestyle factors associated with MI among the Indigenous Shuar of Amazonian Ecuador, who currently experience both a high rate of STH infections [51,52] and rapid but varied levels of MI [8,11]. Previous work indicates that STH infection patterns among the Shuar are influenced by regional differences in MI, such that Shuar living in less market-integrated communities generally exhibit higher levels of parasitism [51,52]. These initial findings suggest that STH infection among the Shuar is influenced by MI-linked lifestyle conditions; however, these studies used only regional comparisons and did not account for specific household-level variables related to MI. Thus, the specific MI factors driving these associations are not well understood. Here, we examine relationships between MI-related changes in household infrastructure and STH infection patterns to facilitate a more nuanced understanding of how lifestyle change impacts parasitic disease patterns.

The objectives and hypotheses of the present study are:

Objective one: To determine whether overall household infrastructure (as measured by a composite estimate of household MI) is significantly related to STH exposure. The term “household infrastructure” reflects number of houses owned, total number of rooms, building materials, latrine type, water source, and presence of electricity. Soil-transmitted helminth infection patterns were assessed using measures of infection status and infection intensity. Infection status reflects whether a participant is infected with at least one STH species, while infection intensity is used as a measure of parasite exposure (beyond a simple measure of infection status). Higher infection intensities reflect continual exposure to sources of infection, which increases the number of mature helminths in the host and the subsequent amount of egg production and ova shed in the feces [53].
*Hypothesis 1*: Soil-transmitted helminth infection risk (odds of being infected with at least one STH species) and infection intensity values will be lower among Shuar reporting higher scores on a composite estimate of household MI (indicating greater overall MI in household infrastructure).
Objective two: To assess the links between STH infection patterns and specific MI factors thought to influence disease transmission (i.e., determine which specific aspects of household infrastructure are strongly linked with STH infection patterns).
*Hypothesis 2*: Soil-transmitted helminth disease risk and infection intensity values will be higher among participants living in houses with dirt floors, no designated latrine, and unfiltered water acquired from a river or stream (i.e., factors likely to facilitate fecal-oral contamination).

## Materials and methods

### Study design

#### Participants

This research was conducted as part of the Shuar Health and Life History Project (SHLHP; shuarproject.org) initiated in 2005. The Shuar are an Indigenous population numbering ~60,000 to 110,000 individuals living in the neotropical lowlands and Andean foothills of southeastern Ecuador and northeastern Peru [11,54–56]. Most Ecuadorian Shuar reside in the Morona-Santiago and Zamora Provinces, in an area that extends from the eastern foothills of the Andes, across the Cordillera de Cutucú, to the upper Amazonian lowlands. Prior to the 1960s, the Shuar resided in dispersed household clusters and lived as semi-nomadic hunter-horticulturalists [55–58]. Shuar subsistence traditionally relied on a mix of slash and burn horticulture, hunting, foraging, and fishing [55,59]. These practices remain important in many Shuar communities but are becoming less common in some areas due to the increasing pace of MI, leading to a greater reliance on market produced foods and wage labor.

Consequently, significant MI heterogeneity is evident within Shuar communities at the household-level [8,11]. For example, there are marked differences in home construction and the ownership of market goods [8,11]. While some Shuar continue to reside in more traditional houses (e.g., constructed of palmwood or cane walls with dirt floors and thatched roofs), others within the same community may live in houses of rough sawn lumber and tin roofs or government-issued cinder block houses. Water source, latrine type, and electrical grid access also vary. This variation likely contributes to different disease risks and health outcomes among individuals even within the same community.

#### Sampling strategy

The present study used a cross-sectional study design, with data collected across six field seasons (2011–14, 2016–17). The first five field seasons took place at the end of the local wet season (July-September), while the final field season (2017) took place at the start of the dry season (September-November; although it was unusually rainy during this particular year). Thus, climatic seasonality is unlikely to explain any systematic differences found in this study, and significant seasonal differences were not observed in STH infection patterns. Participant communities were chosen based on key informant recommendation, snowball sampling, and location (e.g., within reasonable walking distance with equipment from a point accessible by truck or canoe). To help prevent biased sampling, all community members were invited to participate in this study (no willing participants were excluded). The sample included 620 participants (308 females, 312 males, aged 6 months to 86 years), living in 152 households across 10 communities. Sampled communities varied in size and distance from market centers (S1 Table), enabling the assessment of parasitic disease patterns across a wide range of community and household MI levels.

#### Ethics statement

Informed verbal consent was obtained for all adult participants, and parental informed verbal consent and child informed assent obtained for all child participants. The study was approved by the Institutional Review Board of the University of Oregon and authorized by the Federación Interprovincial de Centros Shuar (FICSH). Study methods and procedures were presented to elected community leaders and then to potential participants at open community meetings in which all potential participants could have questions answered. Research was then conducted in accordance with the authorization of elected community leaders upon community consensus. Given a long history of conflicts over land and other rights, asking Shuar participants to sign documents can be considered threatening and lead to suspicion among community members regarding what others were promising with their signatures. Non-literate and partially-literate participants are also common, especially among older participants. To best protect participants, the IRB therefore waived the requirement to obtain documentation of informed consent under 45 CFR 46.117 (c)(2) to allow for a verbal consent process.

### Field and laboratory procedures

#### Market integration measures

Participants completed a structured household interview based on a Material Style of Life (SOL) Index developed by the SHLHP following extensive qualitative research and pre-testing [8,11]. The design of this index and documentation of relevant lifestyle variation was informed by participant observation and living within Shuar communities for extended periods of time. Style of life indices have been successfully used in several anthropological studies to measure lifestyle change in other populations [60,61], and this approach is a useful way to examine MI using variables that most meaningfully reflect lifestyle variation in the study population [8,11]. The Household SOL (H-SOL) index is calculated as a composite score based on different aspects of household infrastructure, including number of rooms, building materials, latrine type, and water source (Cronbach’s α = 0.545; see Table 1 for full list of index variables) [8,11]. Overall, more market-integrated Shuar homes are expected to have higher composite H-SOL scores.

**Table 1:** Shuar Household Style Of Life (H-SOL) index.

Index variables	Score calculation
Houses	Total number
Rooms	Total number
Floor material	0 = dirt; 1 = palmwood; 2 = wood planks; 3 = concrete; 4 = tile
Wall material	0 = palmwood; 1 = mixed; 2 = wood planks; 3 = cinder block
Latrine type	0 = none; 1 = pit; 2 = indoor toilet without water; 3 = outdoor toilet with water; 4 = indoor toilet with water
Water source	0 = river/stream; 1 = well/outdoor pipe; 2 = indoor pipe
Electricity	0 = none; 1 = lights only; 2 = outlets

Variables comprising the Shuar Household Style of Life (H-SOL) index, calculated by summing the scores of reported index variables. Higher total scores reflect greater levels of MI with regards to household infrastructure.

In addition, specific household features hypothesized to influence parasite infection risk were assessed for relationship to STH infection intensity. These characteristics were reported as part of the H-SOL questionnaire and used to calculate categorical variables for floor material (dirt, wood, cement/tile), latrine type (no designated latrine, latrine with no running water, toilet with running water), and water source (river/stream, pipe/well) (Table 2). Each of these three variables were dummy coded before being entered into the models, with “dirt floor”, “no designated latrine”, and “water from a river or stream” serving as the reference groups.

**Table 2:** Descriptive statistics of model variables.

Variable	Mean (SD; range)
Age (years)	20.6 (18.2; 0–86)
Household Style of Life (H-SOL) score	10.5 (3.9; 1–19)
*Ascaris lumbricoides* eggs per gram (EPG) value	6,282.9 (13,633.1; 0–129,336)
*Trichuris trichiura* eggs per gram (EPG) value	142.0 (473.6; 0–6,696)
	**Frequency (%)**
Sex:	
Male	312 (50.3%)
Female	308 (49.7%)
Travel time to nearest market center:	
One hour or more	342 (55.2%)
Under one hour	278 (44.8%)
Community size:	
Small (fewer than 10 houses)	88 (14.2%)
Mid-sized (10–20 houses)	114 (18.4%)
Large (over 20 houses)	418 (67.4%)
Floor material:	
Dirt	122 (19.7%)
Wood	421 (67.9%)
Concrete/Tile	77 (12.4%)
Latrine type:	
No designated latrine	276 (44.5%)
Latrine with no running water	179 (28.9%)
Toilet with running water	165 (26.6%)
Water source:	
River/stream	125 (20.2%)
Pipe/well	495 (79.8%)
Infection status:	
Infected	388 (62.6%)
Not infected	232 (47.4%)

Sample means (with standard deviation and range) or frequency (percent) of model variables, for 620 Shuar participants. For ease of interpretation, untransformed raw values are presented.

#### Stool collection and analysis

Fresh stool samples were collected using previously described methods [51]. A single Kato-Katz smear was prepared from each participant’s fecal sample (by TEG, TJC, JMS, or CJH) within an hour of sample collection [62]. After 30–45 minutes, the smears were examined using 10x and 40x microscopy by experienced observers (either TEG or TJC) and STH infection status, species presence, and species-specific eggs per gram (EPG; indicative of individual infection intensity) of feces were recorded. Infection status was coded as a dichotomous variable (0 = no infection detected; 1 = infection with at least one parasite species detected). Eggs per gram (a continuous variable) was calculated for each distinct STH species by counting the number of eggs present in a 42 mg stool sample and multiplying that egg quantity by 24.

Only first morning stool was collected to limit time of day variation in STH egg shedding. Ascarid (*Ascaris lumbricoides*) and whipworm (*Trichuris trichiura*) infections were predominantly detected, the present study therefore focuses on these two species. In addition, nine tapeworm, one pinworm, and 18 hookworm infections spread across seven communities (with little evidence of clustering within households) were observed. Calculated EPG values were used to determine infection intensity levels based on cutoffs established by Montresor et al. [63]. Specifically, for *A*. *lumbricoides*: light-intensity = 1–4,999 EPG, moderate-intensity = 5,000–49,999 EPG, and heavy-intensity = 50,000+ EPG. For *T*. *trichiura*: light-intensity = 1–999 EPG, moderate-intensity = 1,000–9,999 EPG, and heavy-intensity = 10,000+ EPG.

#### Covariates

Participant characteristics known to influence parasite infection patterns were also included in the statistical models during analysis. Previous work indicates that individual age and sex significantly influence STH infection patterns among the Shuar [52]; age (a continuous variable) and sex were therefore included in the analysis as covariates. Ages were determined by birthdates on government issued identification cards, and cross-checked by informants and existing SHLHP genealogical data, as has been previously described [8,64]. Household size (i.e., the number of individuals living within a household) was also included as a continuous variable to account the role of family size in infection spread.

Community attributes known to influence participant health were also entered in the models as covariates. Increased population sizes and ease of travel to densely populated areas (e.g., market centers) have been linked with increased disease spread [33,65]; community size and approximate travel time from the community to the nearest market center were therefore included during analysis. Community size was entered as a categorical variable (0 = small, under 10 households; 1 = mid-sized, 10–20 households; 2 = large, over 20 households). This community size variable was then dummy coded before being entered into the models, with “small community size” serving as the reference group. Travel time to the nearest market center was entered into the statistical models as a dichotomous variable (0 = one hour or more of travel; 1 = less than an hour of travel). The travel time variable reflected access to market centers and medical care. In the present study, every community located less than one hour from a market center also had easy access (i.e., travel time under 30 minutes) to a regularly staffed medical clinic.

### Statistical analyses

Data analyses were conducted using Stata 14. The *Ascaris lumbricoides* EPG variable was non-normally distributed, with skewness of 3.51 (SE = 0.098) and kurtosis of 17.19 (SE = 0.196). Likewise, *T*. *trichiura* EPG values were non-normally distributed, with skewness of 7.56 (SE = 0.098) and kurtosis of 79.17 (SE = 0.196). These values were natural log-transformed to achieve normal distributions (skewness values within ±1, kurtosis values within ±2). Extreme outliers were not excluded from analyses due to the overdispersed nature of EPG values; removing these outliers would remove participants with high infection intensities and therefore data points of interest to this specific study. Multicollinearity was not detected between any variables; all VIF values were in an acceptable range of 1.00–1.70 [66].

To test the hypotheses, two models were run for each outcome variable of interest (STH infection status, *A*. *lumbricoides* infection intensity, and *T*. *trichiura* infection intensity):

- Hypothesis 1: The first set of models tested whether odds of STH infection and species-specific infection intensity variation were inversely related to a composite estimate of household MI.
- Hypothesis 2: The second set of models tested if odds of STH infection and species-specific infection intensity variation were significantly associated with three SOL factors hypothesized to drive STH exposure (floor material, latrine type, and water source).

Given that two sets of analyses were run for each STH-related dependent variable (i.e., to test the two hypotheses for infection status, *A*. *lumbricoides* infection intensity, and *T*. *trichiura* infection intensity), a Bonferroni correction was applied, and results were considered significant at *p* < 0.025.

#### Testing associations between household MI and infection status

Multilevel models were used to test associations between infection status and household characteristics; these models captured the interdependence evident in the dataset (e.g., individuals nested within households) [67]. Specifically, mixed effects logistic regressions were conducted to test associations between infection status (a dichotomous variable) and the H-SOL variables. Participant age and sex were entered at Level-1 of the models as fixed effects, while household size, community size, travel time to the nearest market center, and the H-SOL variables of interest were entered at Level-2; all variables were entered simultaneously. No significant interaction was evident between participant age and sex in any model tested (all *p* > 0.05).

Three-level models (individuals nested in household nested in communities) were not used to test the hypotheses. An important issue in multilevel analysis is ensuring that the sample size at each level is sufficient for accurate model estimation; small sample size at the highest level of the model may compromise analysis accuracy (e.g., by leading to biased standard error estimates) [68]. Two-level models were therefore fit, due to the relatively small number of communities sampled (n = 10), and the small Intraclass Correlation Coefficient (ICC; a measure of the proportion of total variance in EPG values accounted for by clustering at each level of the model) values associated with Level-3. Each Level-3 ICC value was below 10%, a common cut-off used to determine whether the level accounts for a meaningful amount of total model variance [69]. Moreover, some of the communities included in this study were smaller than the others, resulting in relatively small community samples (i.e., 10–18 people), which may also lead to biased model estimates.

Participant community was instead controlled in the analyses by including the two fixed effects variables (entered at Level-2) reflecting community size and travel time to the nearest market center. The associations between these community characteristic variables and the STH infection variables were consistent across both two- and three-level models (where the community variables were entered at Level-3), suggesting that the community characteristic findings in the two-level models are meaningful.

Preliminary model-fit testing was performed to assess the need for multilevel models and to determine which variables should be included in the final model. Three models, increasing in complexity, were fit for the infection status variable. Likelihood ratio tests were conducted between each successive model to test whether the expanded model better fit the data. The models assessed are as follows:

- **Model 1**: a one-level null model, containing no predictors.
- **Model 2**: a two-level random intercept null model adding a second level (individuals nested in households) to Model 1.
- **Model 3**: a two-level model adding all fixed predictors (entered simultaneously) to Model 2.

Based on the results of these analyses, Model 3 was selected as the best fit model. However, not all covariates contributed meaningfully to the final, two-level model. Adding household size to the final two-level model (including all fixed effect predictors) did not significantly improve model fit (all *p* > 0.15). Furthermore, ten participants were missing these data; sensitivity analyses indicated that these values were missing at random. The household size variable was therefore dropped from all models. No other variables were missing data.

#### Testing associations between household MI and infection intensity

Similar to other studies using helminth infection intensity counts [70], overdispersion and zero-inflation were apparent in the dataset. Preliminary tests comparing zero-inflated models to standard Poisson models indicated that zero-inflated negative binomial (ZINB) regression models better fit the data (i.e., had lower AIC and BIC values). Zero-inflated negative binomial regression models were therefore used to test relationships between parasite EPG variation and the H-SOL variables. These models account for the possibility of zero values in the dataset being generated through more than one process, meaning that some of the zero values represent “true zeroes” (i.e., people who are truly not infected), while others may reflect methodological failures to measure infection intensity. For instance, infected participant stool samples may not have contained helminth ova if they were in the pre-patent phase of infection (i.e., were infected, but the parasites had not yet matured to the point of producing diagnostic eggs shed in stool) or if helminth ova were not evident in that specific stool sample due to uneven shedding. These factors would lead to participants being incorrectly assigned an infection intensity count of zero. Zero-inflated negative binomial regression models account for this complication in the data. This approach is consistent with previous helminth research [e.g., 70–72].

Specifically, ZINB analysis fits two separate models and then combines them. The first is a logit model which predicts whether a participant should be in the “true zero” group. In other words, this part of the model estimates if predictors significantly impact whether participants are correctly identified as not infected (with an accurate EPG value of zero, a “true zero”), as opposed to being incorrectly classified as having an EPG value of zero when they are in fact infected. Second, a negative binomial model predicts the infection intensity counts for participants that are not “true zeroes” (i.e., are in fact infected). The two models are then combined, and a single output provided. The confounders (sex, age, travel time to the nearest market center, and community size) and relevant H-SOL variables were entered in the negative binomial model. Meanwhile, participant sex and age were entered in the logit model, to account for influence of these individual characteristics in shaping both helminth ova shedding patterns and the likelihood of being in the pre-patent phase of infection. The nested random effects were included in the ZINB models by using the vce(cluster) command. This command conservatively alters the way standard errors are calculated and removes the assumption of independent observations, requiring instead that each “cluster” is independent. Clusters were defined as participants within households.

## Results and discussion

### Descriptive statistics

**Fig 1:**
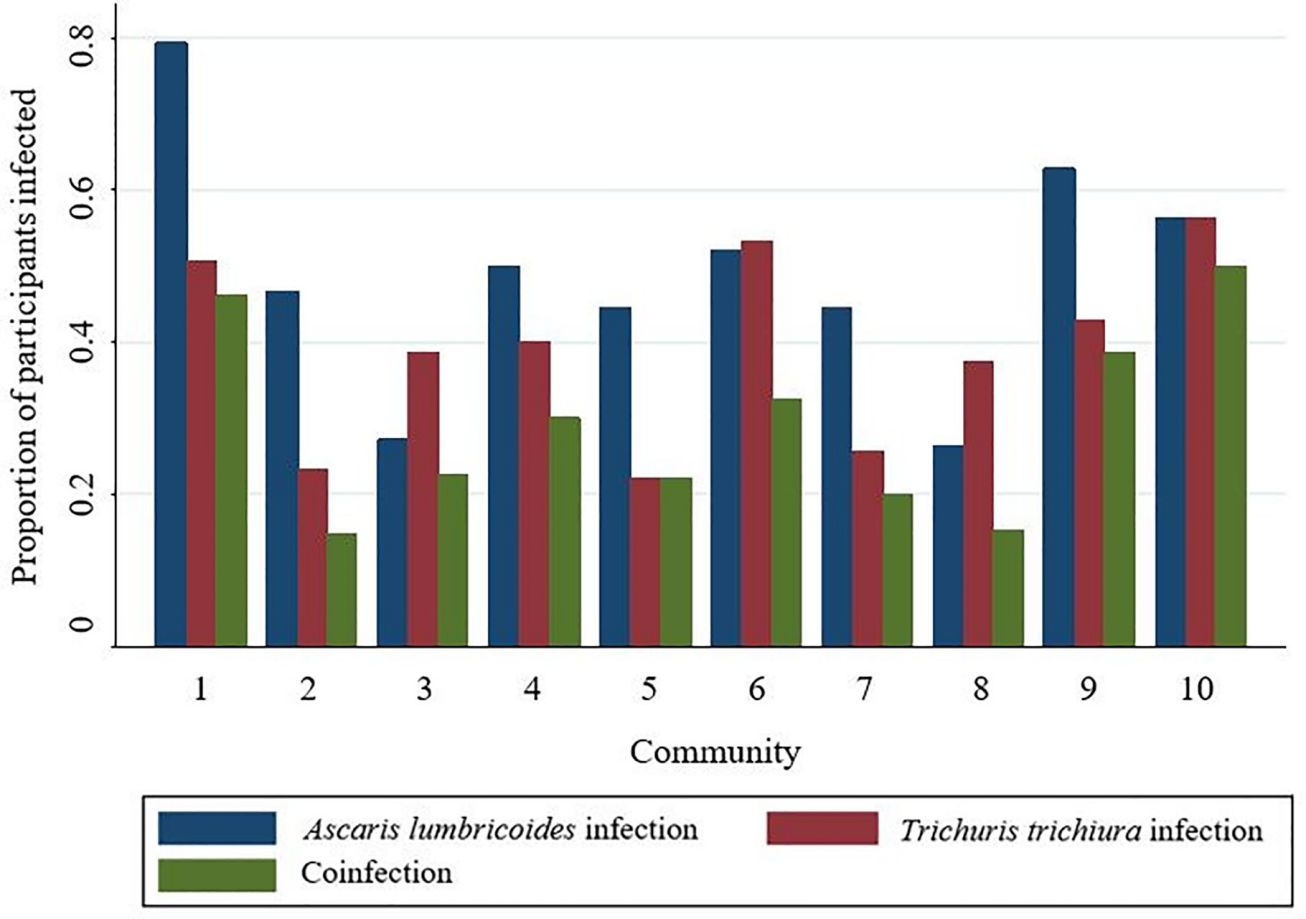
Infection prevalence rates. Proportion of participants by community infected with *A*. *lumbricoides*, *T*. *trichiura*, or coinfected with both species. See S1 Table for community descriptions. Communities are listed in order of median household style of life scores, which serves as an approximation of community market integration level. Community median style of life scores are presented from lowest to highest (i.e., community 1 has the lowest median score, while community 10 has the highest).

**Fig 2:**
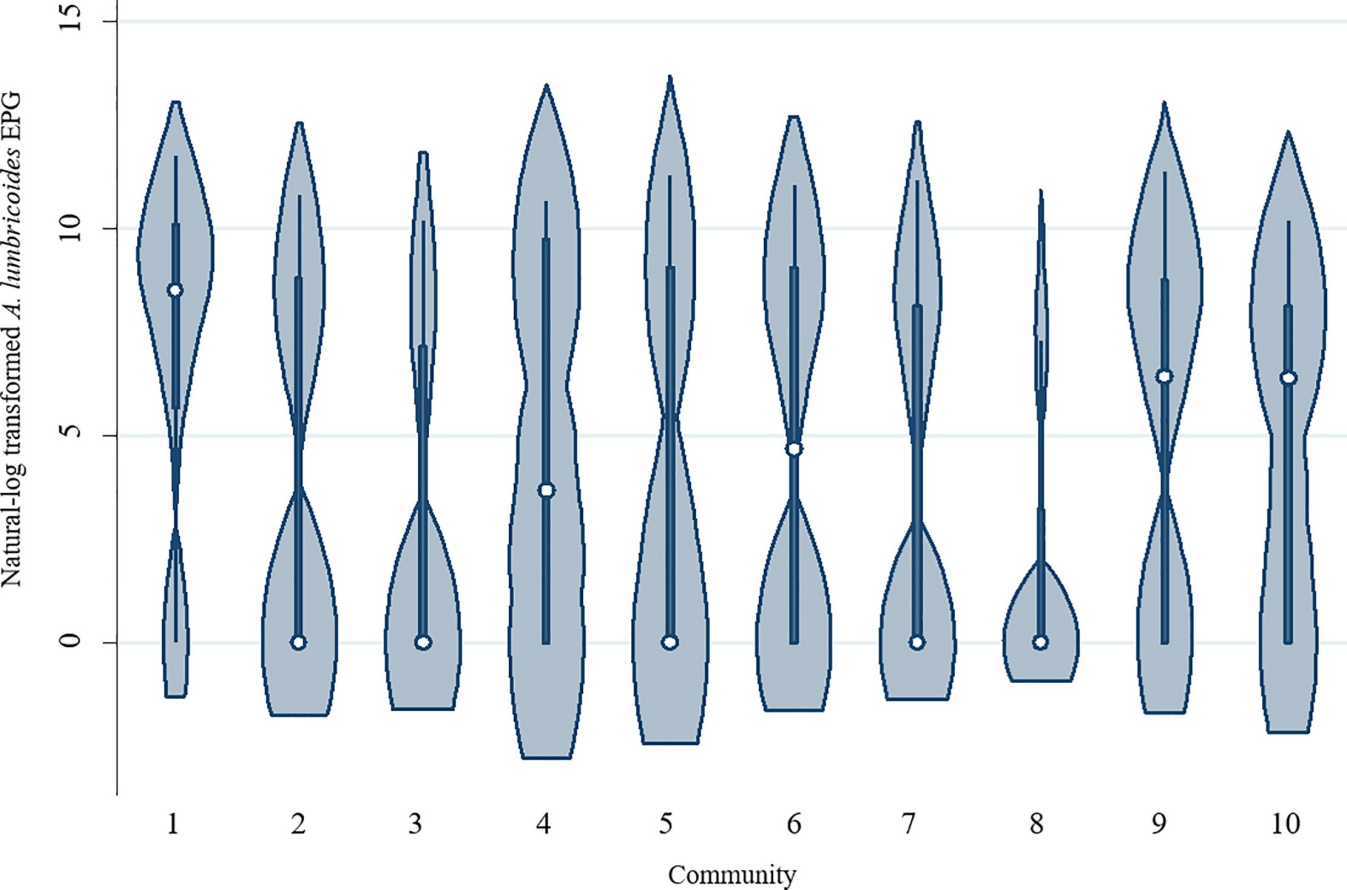
*A*. *lumbricoides* infection intensity values by community. Violin plots depicting *A*. *lumbricoides* eggs per gram (EPG) infection intensity values by community. The white circle marks the community median, the box indicates community interquartile range, with whiskers extending to the upper and lower-adjacent values. Overlaid shape reflects the probability density of the data at different EPG values. Communities are listed in order of median household style of life scores, which serves as an approximation of community market integration level. Community median style of life scores are presented from lowest to highest (i.e., community 1 has the lowest median score, while community 10 has the highest).

**Fig 3:**
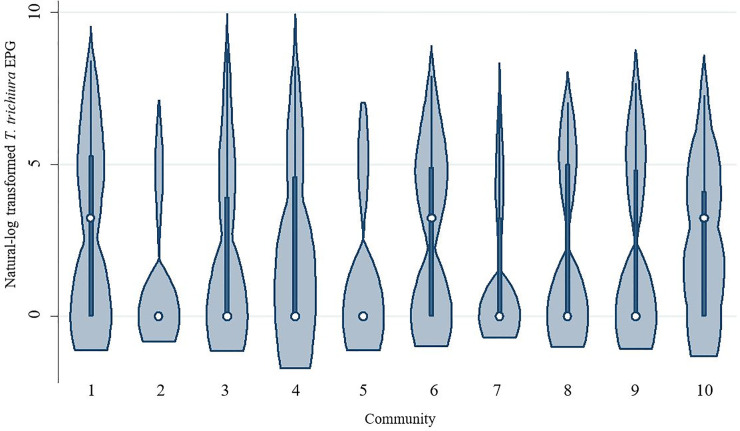
*T*. *trichiura* infection intensity values by community. Violin plots depicting *T*. *trichiura* eggs per gram (EPG) infection intensity values by community. The white circle marks the community median, the box indicates community interquartile range, with whiskers extending to the upper and lower-adjacent values. Overlaid shape reflects the probability density of the data at different EPG values. Communities are listed in order of median household style of life scores, which serves as an approximation of community market integration level. Community median style of life scores are presented from lowest to highest (i.e., community 1 has the lowest median score, while community 10 has the highest).

Table 2 presents descriptive statistics for all variables included in the models. Males and females comprised roughly equal parts of the sample, and mean participant age was relatively young (20.6 years old). The composite measure of household infrastructure exhibited a wide range, supporting the premise that a large amount of variability in household MI is present in the sample. Most participants reported living in homes with floors made of wood (67.9%) and reliance on water from a well or piped from a spring (79.8%), while nearly half of all participants (44.5%) indicated they did not use a designated latrine. A little over half of all participants (55.2%) lived within an hour of a market center, and most participants (67.4%) lived in a large community (with 20+ houses). Of the 620 participants, 62.6% were infected with at least one of the two STH species (*A*. *lumbricoides* and *T*. *trichiura*), and 26.8% were co-infected, although infection prevalence varied across communities (Fig 1). The distribution of EPG values also varied by community, with some communities displaying comparatively high EPG median values due to more community members exhibiting relatively high STH infection intensity values (Figs 2 and 3).

Based on WHO standards [63], of the 301 participants infected with *A*. *lumbricoides*, 47.5% exhibited light-intensity infections, 48.5% had moderate-intensity infections, and 4.0% exhibited heavy-intensity infections. In contrast, of the 231 participants infected with *T*. *trichiura*, 90.0% had light-intensity infections, 10.0% exhibited moderate-intensity infections, and no individuals had heavy-intensity infections.

### The relationship between measures of MI and STH infection status

#### Hypothesis 1: Level of MI (as reflected by H-SOL score) will be inversely associated with odds of STH infection

A two-level model was used to examine how a composite measure of household infrastructure was related to odds of being infected with at least one STH species (Table 3). Intraclass correlation coefficients (ICC) values were calculated from null models containing no predictors. These values indicate that roughly 25.6% of the odds of infection was accounted for by between-household (Level-2) differences, while the remaining 74.4% was explained by between-individual (Level-1) differences. The results from the final two-level model indicate that participant age was a significant predictor of infection status (OR = 0.988, SE = 0.005, *p* = 0.020), implying that older individuals had lower odds of being infected. Travel time to nearest market center was also a significant predictor (OR = 2.13, SE = 0.592, *p* = 0.007), with participants living within an hour of a market center displaying increased odds of infection compared to those living an hour or more away. The composite measure of household infrastructure was also significantly related to infection risk (OR = 0.886, SE = 0.033, *p* = 0.001), such that participants living in houses with a higher composite measure of household infrastructure–indicative of more market integrated aspects of household infrastructure–exhibited lower odds of infection.

**Table 3:** Associations between household characteristics and infection status.

	*Infection status*^*a*^: *Model with Composite H-SOL Score*	*Infection status*^*a*^: *Model with Specific Household Characteristics*
***Fixed Effects***		
**Intercept**	3.49 (1.56; 1.45–8.39)*	2.91 (1.24; 1.26–6.70)*
**Age**	0.988 (0.005; 0.978–0.998)*	0.988 (0.005; 0.978–0.999)
**Sex**^**b**^	0.877 (0.167; 0.605–1.27)	0.875 (0.166; 0.603–1.27)
**Travel time to nearest market center**^**b**^	2.13 (0.592; 1.23–3.67)*	1.97 (0.683; 0.996–3.88)
**Community size**^**b**^:		
** Mid-sized (10–20 houses)**	2.43 (1.09; 1.01–5.84)	2.83 (1.44; 1.04–7.69)
** Large (over 20 houses)**	1.89 (0.682; 0.936–3.84)	2.21 (0.923; 0.976–5.01)
**Household Style of Life (H-SOL) score**	0.886 (0.033; 0.824–0.952)*	---
**Floor material**^**b**^:	---	
** Wood**	---	0.410 (0.146; 0.204–0.824)*
** Concrete/Tile**	---	0.424 (0.234; 0.143–1.25)
**Latrine type**^**b**^:	---	
** Latrine with no running water**	---	0.693 (0.214; 0.379–1.27)
** Toilet with running water**	---	0.770 (0.288; 0.370–1.60)
**Water source**^**b**^:	---	
** Pipe/well**	---	0.686 (0.267; 0.319–1.47)
***Random Effects***		
** Household Intercept**	0.781 (0.313; 0.356–1.71)	0.741 (0.309; 0.327–1.68)

Multilevel logistic regression models assessing associations between the Household Style of Life (H-SOL) index (composite model) or select household characteristics (specific model) and helminth infection risk, for individuals nested within households. Odds ratios (for infection status) are provided with standard errors and 95% CI for every variable included in each respective model. Results are statistically significant at

* = *p* < 0.025

** = *p* < 0.001.

^a^A dichotomous variable (0 = not infected, 1 = infected with at least one parasite species).

^b^Reference groups = male, travel time of one hour or more (vs. travel time of less than one hour), small (fewer than 10 houses), dirt floor, no designated latrine, water from a river/stream.

#### Hypothesis 2: Odds of STH infection will be higher among participants living in houses with dirt floors, no designated latrine, and water use from a river or stream

Additional analyses tested whether specific aspects of household infrastructure were significant predictors of infection risk (Table 3). Participant age, sex, travel time to nearest market center, community size, latrine types, and water source were all non-significant predictors. The only significant predictor in the model was floor material (OR = 0.410, SE = 0.146, *p* = 0.012), suggesting that participants living in households with floors made of wood had significantly lower odds of infection compared to individuals living in homes with dirt floors.

### Associations between measures of MI and STH infection intensity

#### Hypothesis 1: Level of MI (as reflected by a composite measure of household infrastructure) will be inversely associated with STH infection intensity (measured using EPG values)

Zero-inflated negative binomial (ZINB) regression models were used to test associations between a composite measure of household infrastructure and parasite EPG variation (Table 4). The results indicate that participant sex, travel time to nearest market center, and community size were not significantly associated with *A*. *lumbricoides* EPG variation (Table 4). However, age was a significant predictor of *A*. *lumbricoides* EPG variation (B = -0.003, z = -4.11, *p* < 0.001), suggesting older individuals had lower infection intensities. Likewise, the composite measure of household infrastructure was a significant predictor of *A*. *lumbricoides* EPG variation (B = -0.011, z = -3.53, *p* < 0.001), indicating that participants living in houses exhibiting greater overall levels of MI exhibited lower *A*. *lumbricoides* infection intensities as hypothesized. Neither age nor sex were significant predictors in the logit part of the combined model.

**Table 4:** Associations between Household Style of Life (H-SOL) scores and helminth infection intensity variation.

	*Ascaris lumbricoides EPG*^*a*^	*Trichuris trichiura EPG*^*a*^
***Negative binomial model***		
**Intercept**	2.27 (0.040; 2.19–2.35)**	1.74 (0.071; 1.60–1.88)**
**Age**	-0.003 (0.001; -0.004-(-0.002))**	-0.007 (0.001; -0.008-(-0.005))**
**Sex**^**b**^	0.015 (0.023; -0.031–0.061)	-0.040 (0.034; -0.106–0.027)
**Travel time to nearest market center**^**b**^	0.019 (0.029; -0.038–0.076)	0.117 (0.044; 0.030–0.203)*
**Community size**^**b**^:		
** Mid-sized (10–20 houses)**	0.019 (0.041; -0.061–0.099)	0.142 (0.072; 0.001–0.284)
** Large (over 20 houses)**	0.013 (0.033; -0.051–0.078)	0.072 (0.062; -0.049–0.193)
**Household Style of Life (H-SOL) score**	-0.01 (0.003; -0.017-(-0.005))**	-0.016 (0.006; -0.028-(-0.004))*
***Logit model***		
**Intercept**	-0.107 (0.145; -0.391–0.177)	-0.145 (0.153; -0.444–0.154)
**Age**	0.005 (0.004; -0.004–0.013)	0.023 (0.005; 0.013–0.034)**
**Sex**^**b**^	0.140 (0.161; -0.176–0.456)	0.405 (0.171; 0.069–0.741)*
**Dispersion parameters of the model**		
**/lnalpha**	-18.31 (0.042;	-121.00 (0.077;
	-18.39-(-18.22))**	-121.15-(-120.85)**
**alpha**	1.12e-08 (4.72e-10;	2.83e-53 (2.17e-54;
	(1.03e-08)–(1.22e-08))	(2.44e-53)–(3.29e-53))

Zero-inflated negative binomial (ZINB) regression models assessing associations between the Household Style of Life (H-SOL) index and helminth eggs per gram (EPG) variation, accounting for model clustering (i.e., of individuals within households). Negative binomial regression coefficients are provided with standard errors and 95% CI for each variable included in the model. Results are statistically significant at

* = *p* < 0.025

** = *p* ≤ 0.001.

^a^All EPG values were natural log-transformed-transformed to achieve normal distributions.

^b^Reference groups = male, travel time of one hour or more (vs. travel time of less than one hour), small (fewer than 10 houses), dirt floor, no designated latrine, water from a river/stream.

Slightly different patterns emerged in the *T*. *trichiura* multilevel model (Table 4). Participant age (B = -0.007, z = -6.55, *p* < 0.001) and travel time to the nearest market center (B = 0.117, z = 2.65, *p* = 0.008) were both significant predictors of *T*. *trichiura* EPG, such that older individuals displayed lower EPG values and participants living within an hour of a market center exhibited higher infection intensities than those living an hour or more away. The composite measure of household infrastructure was also significantly related to *T*. *trichiura* EPG as predicted (B = -0.016, z = -2.71, *p* = 0.007), signaling that individuals living in houses with greater overall levels of MI displayed lower *T*. *trichiura* infection intensities. Interestingly, both age (B = 0.023, z = 4.42, *p* < 0.001) and sex (B = 0.405, z = 2.36, *p* = 0.018) were significant predictors in the logit part of the combined model, suggesting that older individuals and females (vs. males) were more likely to be “true zeroes” (e.g., be accurately classified as having an EPG value of zero, as opposed to being a false negative).

#### Hypothesis 2: Soil-transmitted helminth infection intensities will be higher among participants living in houses with dirt floors, no designated latrine, and using unfiltered water

Additional ZINB regression analyses were conducted to assess the relationship between the three specific household characteristic variables (floor material, latrine type, and water source) and parasite EPG value variation (Table 5). Results from this second set of analyses indicated that participant sex, travel time to nearest market center, community size, latrine type, and water source were not significantly associated with *A*. *lumbricoides* EPG variation. Conversely, age was a significant predictor (B = -0.003, z = -3.75, *p* < 0.001), indicating that older individuals had lower infection intensities. In addition, water source was a significant predictor (B = -0.096, z = -3.29, *p* = 0.001), signaling that participants using water from a well or piped from a spring had lower *A*. *lumbricoides* infection intensities compared to participants reliant on water from a river or stream, as predicted. However, latrine type and floor material did not significantly contribute to *A*. *lumbricoides* infection intensity. Similarly, neither age nor sex were significant predictors in the logit part of the combined model.

**Table 5:** Associations between specific aspects of household infrastructure and helminth infection intensity variation.

	*Ascaris lumbricoides EPG*^*a*^	*Trichuris trichiura EPG*^*a*^
***Negative binomial model***		
**Intercept**	2.23 (0.036; 2.16–2.30)**	1.72 (0.068; 1.59–1.85)**
**Age**	-0.003 (0.001; -0.004-(-0.001))**	-0.006 (0.001; -0.008-(-0.005))**
**Sex**^**b**^	0.019 (0.023; -0.027–0.065)	-0.029 (0.035; -0.096–0.039)
**Travel time to nearest market center**^**b**^	-0.024 (0.040; -0.101–0.054)	0.073 (0.052; -0.029–0.174)
**Community size**^**b**^:		
** Mid-sized (10–20 houses)**	0.057 (0.056; -0.053–0.167)	0.158 (0.079; 0.003–0.313)
** Large (over 20 houses)**	0.050 (0.038; -0.026–0.125)	0.078 (0.068; -0.056–0.211)
**Floor material**^**b**^:		
** Wood**	-0.028 (0.029; -0.085–0.028)	-0.133 (0.052; -0.235-(-0.031))*
** Concrete/Tile**	0.008 (0.059; -0.107–0.124)	-0.121 (0.075; -0.268–0.027)
**Latrine type:**		
** Latrine with no running water**	-0.013 (0.032; -0.075–0.050)	0.061 (0.044; -0.025–0.146)
** Toilet with running water**	0.001 (0.043; -0.084–0.085)	0.062 (0.047; -0.030–0.154)
**Water source**^**b**^:		
** Pipe/well**	-0.096 (0.029; -0.153-(-0.039))**	-0.080 (0.048; -0.175–0.014)
***Logit model***		
**Intercept**	-0.107 (0.145; -0.391–0.177)	-0.145 (0.152; -0.444–0.154)
**Age**	0.005 (0.004; -0.004–0.013)	0.023 (0.005; 0.013–0.034)**
**Sex**^**b**^	0.140 (0.161; -0.176–0.456)	0.406 (0.171; 0.070–0.742)*
**Dispersion parameters of the model**		
**/lnalpha**	-18.30 (0.040;	-193.88 (0.062;
	-18.38-(18.22))**	-194.00-(-193.76))**
**alpha**	1.12e-08 (4.50e-10;	6.28e-85 (3.91e-86;
	(1.04e-08)–(1.22e-08))	(5.56e-85)–(7.09e-85))

Zero-inflated negative binomial (ZINB) regression models assessing associations between select household characteristics and helminth eggs per gram (EPG) variation, accounting for model clustering (i.e., of individuals within households). Negative binomial regression coefficients are provided with standard errors and 95% CI for each variable included in the model. Results are statistically significant at

* = *p* < 0.025

** = *p* ≤ 0.001.

^a^All EPG values were natural log-transformed-transformed to achieve normal distributions.

^b^Reference groups = male, travel time of one hour or more (vs. travel time of less than one hour), small (fewer than 10 houses), dirt floor, no designated latrine, water from a river/stream.

The results of the *T*. *trichiura* analyses varied slightly from the *A*. *lumbricoides* analyses (Table 5). Age (B = -0.006, z = -6.86, *p* < 0.001) was significantly associated with *T*. *trichiura* EPG variation, signifying that older individuals had lower *T*. *trichiura* infection intensities. Neither latrine type nor water source was significantly associated with *T*. *trichiura* infection intensity. However, floor material was a significant predictor (B = -0.133, z = -2.56, *p* = 0.010), with participants living in households with wood floors displaying significantly lower *T*. *trichiura* infection intensities compared to individuals living in houses with dirt floors. Again, both age (B = 0.023, z = 4.43, *p* < 0.001) and sex (B = 0.406, z = 2.37, *p* = 0.018) were significant predictors in the logit part of the combined model, suggesting that older individuals and females were more likely to be “true zeroes”.

## Discussion

The current study documents relationships between STH infection status, infection intensity, and MI level, using characteristics related to household infrastructure among Indigenous Amazonian Shuar. The results presented here support the first hypothesis. As predicted, individuals from more market integrated households (i.e., scoring highly on a composite measure of household infrastructure) displayed significantly lower odds of infection, as well as lower *A*. *lumbricoides* and *T*. *trichiura* infection intensity values. However, the study provides mixed support for the second hypothesis. Individuals living in households with floors of wood (as opposed to dirt floors) exhibited significantly lower odds of infection and lower infection intensities for *T*. *trichiura*, while participants using water from a well or piped from a spring (as opposed to from a river or a stream) had lower *A*. *lumbricoides* infection intensities, as hypothesized. Yet, latrine type was unexpectedly not a significant predictor of infection intensity for either STH species.

### Household style of life (H-SOL) and infection patterns

The composite measure of household infrastructure was a significant predictor of parasite infection status and intensity (for both *A*. *lumbricoides* and *T*. *trichiura*), implying that MI-linked changes in household infrastructure offered some protection from STH infection. This is unsurprising given that this household composite measure was designed to capture aspects of MI known to impact exposure to disease sources directly. Furthermore, accounting for clustering within households (i.e., adding participant household at Level-2 during analysis) significantly improved model fit in the infection status logistic regression analysis. These findings suggest STH exposure within the household plays an important role in parasitic disease risk variation, supporting the idea that increased household MI may help reduce parasitic disease exposure for the two most prevalent STH species observed among the Shuar.

However, it is important to note that degree of household MI varied within communities. While certain communities did exhibit patterns of higher or lower H-SOL scores overall, some communities exhibited a greater range of H-SOL scores (S1 Table). For instance, the least market integrated community (as calculated by community median household style of life scores) exhibited H-SOL scores ranging between 2–6, while the most market integrated community displayed a larger range (from 7–19). This variation is likely due to a range of factors, including personal preferences in house design and function (e.g., a preference for more traditional vs. more market integrated building materials), access to traditional building materials, or inability to afford certain household characteristics. It is due to this degree of variation between homes that market integration was primarily assessed at the household level, rather than the community or regional level [51,52].

Still, despite household variation within communities, the protective effects of increasing MI within participant households consistently appear to decrease overall STH exposure. Yet, moderate- and heavy-intensity infections were still prevalent in this sample, especially among participants infected with *A*. *lumbricoides* (~52.5% of *A*. *lumbricoides* infections were classified as moderate or heavy). These values are consistent with previous work conducted among the Shuar, which found that Shuar exhibit relatively high infection intensities compared to other subsistence-based and rural populations [50]. Reducing rates of reinfection should therefore be a priority for interventions aiming to reduce STH burden in this group.

The provision of a one-time antihelminth treatment–a common public health intervention–is only a short-term solution to a recurring problem caused by high rates of reinfection. The results presented here instead suggest that investing in household construction and clean water systems might be a more effective way to prevent continual exposure to sources of STH infection. Efforts to prevent reinfection could have long lasting benefits, especially among children. Previous work has documented high rates of stunted growth (~40%) among Shuar children [73], likely due in part to constrained energy budgets and the diversion of energetic resources to immune activity among children with persistent parasitic infections [74–76]. Efforts to prevent reinfection after treatment could therefore improve not only immediate health outcomes but long-term development processes. While it is not typically possible to reconstruct entire houses to avoid STH reinfection, identifying the specific household characteristics most strongly linked with parasitic disease risk can help inform the design of ongoing government sponsored housing programs, water system development, and other intervention programs to better target key sources of infection.

### Household characteristics and infection patterns

The second set of models tested whether specific aspects of household infrastructure (floor material, latrine type, and water source) were significantly related to STH infection status or intensity. The models documented some meaningful associations between two specific household characteristics and STH infection patterns, suggesting that water source and floor material are especially important sources of STH exposure among the Shuar. These findings are consistent with known infection transmission pathways, whereby STH eggs present in dirt on the ground or water may result in (re)infection through hand-to-mouth transmission and the contamination of food items [41]. Future interventions should therefore consider the important role floor material and water source play in STH transmission. Constructing household floors of wood (in place of dirt) may be an effective and cost-efficient way to disrupt the spread of initial helminth infections and reduce *T*. *trichiura* endemic exposure, while the construction of wells or pipes may help reduce *A*. *lumbricoides* loads in this population. In addition, easy access to wells or piped spring water may promote sanitary behaviors such as hand washing, which may also reduce the spread of STH infection.

While this level of financial investment in household infrastructure may not be possible for many individual Shuar families, there have been ongoing local efforts in Ecuador to address widespread helminth infection. For example, a report on the results of our STH prevalence and intensity research was presented to Federación Interprovincial de Centros Shuar for use in their solicitation for additional healthcare funds from the Ecuadorian government and NGOs. Thus, the results presented here can be used in similar future advocacy efforts; specifically, to bolster and/or solicit government and NGO programs to invest in additional housing infrastructure across Shuar communities and to focus these efforts on improving water quality and encouraging the use of wood in floor construction. The study findings presented here may also be useful to individual Shuar. Due to ongoing MI, many Shuar are experiencing rapid changes in lifestyle, including increased wage labor (providing some families with disposable income) and access to new household building materials. These results may encourage individuals with available financial and material resources to invest in household infrastructure–especially water quality and floor material–as a good first step toward reducing disease exposure. Steps have been taken to share study data with Shuar participants. Specifically, participants were informed of their infection status and infected individuals were offered medical treatment at no cost through collaborations with local health providers. Summarized results are also provided in community presentations, highlighting local factors linked with infection risk.

Interestingly, although floor material and water source were related to STH infection patterns as hypothesized, latrine type was not significantly associated with STH infection status or intensity (for either STH species). Latrine type may not have been a significant predictor due to a general lack of sewage or septic systems and sanitary bathrooms with running water across all communities in the sample, factors that are known to decrease risk of infection transmission [77,78]. The vast majority of participants (~98%) used latrines located outside of the house, mostly without water, with nearly half reporting no designated latrine and only 10 participants indicating they had toilets with water inside their homes (which were government provided and was typically not connected to a water system). Thus, a shared lack of sanitation may explain why this variable failed to contribute significantly to STH infection variability in the present study.

### Infection pattern differences between parasite species

The two species of interest (*A*. *lumbricoides* and *T*. *trichiura*) exhibited similar patterns with regards to the first study hypothesis: infection risk and intensities for both species were inversely related with a composite measure of household infrastructure. Still, differences in infection patterns were apparent between *A*. *lumbricoides* and *T*. *trichiura*. Specifically, water source was a significant predictor for *A*. *lumbricoides* infection intensity, while floor material was a more important determinant of *T*. *trichiura* infection intensity. These findings suggest that the concentration of helminth eggs within the environment vary by species, perhaps due to differences in egg production and infectivity patterns. For instance, *Ascaris lumbricoides* is known to infect people at a relatively low dose of exposure and produces eggs that are highly resistant to harsh environmental conditions [41,79,80]. Moreover, *A*. *lumbricoides* is among the most prolific egg layers of the gut nematodes; a single female can produce ~200,000 eggs per day (compared to 3,000–5,000 eggs per day from a single *T*. *trichiura* female) [41,79,80]. Environmental sampling of soil and water sources in Shuar communities is required to quantify these differences.

In addition, some species differences were apparent among the covariates entered in the models. As has previously been documented among the Shuar and other populations [43,52,81–83], age seems to be consistently related to infection patterns, with younger individuals exhibiting higher odds of infection and higher infection intensities for both species. However, travel time to the nearest market center was significant in only the *T*. *trichiura* household style of life model. Specifically, participants living in communities located within an hour of the nearest market center exhibited higher *T*. *trichiura* infection intensities compared to participants living in communities located an hour or more away. These findings suggest that certain community characteristics associated with greater MI levels (i.e., increased connectivity between towns) may increase *T*. *trichiura* (but not *A*. *lumbricoides*) disease transmission. These results are consistent with work suggesting that parasitic disease spread is facilitated by the increased movement of goods, livestock, and contaminated food items in highly populated areas [84].

Species differences in infection intensity were also apparent in the logit part of the ZINB models. Age and sex were not significant predictors in this part of the model for *A*. *lumbricoides* but were both significant in the *T*. *trichiura* models. These findings suggest that older individuals and females are more likely to be “true zeroes” (as opposed to being accidently misclassified as not infected when they actually were infected with *T*. *trichiura*). This finding is consistent with the other study results indicating an inverse relationship between age and infection intensity; however, it is interesting that sex is significant in the logit model when it was not a significant predictor in the negative binomial part of the combined model. Thus, while infection intensity does not appear to significantly vary between infected males and females, the logit results could suggest that females are less likely to be infected with *T*. *trichiura* in the first place, as has been previously documented among the Shuar and other populations [52,82,85]. This sex difference may result from gender-based divisions of labor. For example, Shuar men tend to travel more (increasing risk of disease exposure from other communities) and may encounter livestock more frequently (elevating zoonotic disease risk). However, additional data collection is needed to test these disease transmission pathways.

Interestingly, while increasing MI within participant households (as reflected by H-SOL score, floor materials, and water sources) was significantly linked with decreased STH infection risk and intensity, being located closer to a market center–a factor indicative of greater MI within communities–appears to have the opposite effect on *T*. *trichiura* infection. These conflicting findings highlight the complex effects of MI on parasitic disease, perhaps explaining similar heterogenous effects of MI on growth and cardiovascular health among the Shuar [8,11]. Some aspects of MI (e.g., improving water quality or constructing houses with wood floors in place of dirt) appear to reduce parasitic disease burdens, while others (e.g., greater connectivity between towns) may instead exacerbate disease spread for some parasite species. Future neglected tropical disease research should consequently adopt a nuanced perspective when considering the effects of MI on infection exposure. Acknowledging the multifaceted effects of MI on disease spread will be increasingly important as MI continues at an accelerating rate among populations worldwide.

### Study limitations

This study has several important limitations. First, only one stool sample was collected from each participant, which allowed for a larger sample size but may have resulted in the misclassification of some infected participants as uninfected. Eggs per gram measures vary substantially throughout the day, and multiple stool samples are ideal for quantifying infection status. To remedy this, we only accepted first morning stool to limit variation based on time of day and ran ZINB models to account for participants being incorrectly assigned an infection intensity count of zero. Multiple samples per participant, however, would have increased the accuracy of infection intensity calculations.

Second, fecal samples were analyzed using the Kato-Katz method, which is useful for field studies but not ideal for detecting certain types of STH infection (e.g., hookworm infections) [86,87]. Still, the relatively low levels of anemia present among Shuar suggest that hookworm infections may not be especially prevalent in this population [51].

Third, the data collected here is cross-sectional in nature. It is therefore not possible to determine how individual infection intensities may vary across time, making it difficult to definitively determine whether higher helminth infection intensities are the result of continual exposure to infection–as hypothesized here–or due to immunosuppression preventing the clearance of heavy parasite loads. While participants were asked about their medical history and current symptoms during data collection to identify any pre-existing conditions that may impact parasite infection patterns, it is possible that participants with undiagnosed conditions or dysregulated immune activity may exhibit high intensity infections without being chronically exposed to sources of infection. Longitudinal data are needed to assess how changes in immune activity are related to parasite load over time.

Finally, the present analyses did not consider participant antihelminth medication use. Many study participants failed to report these data (n = 124, 20% of the sample); this variable was therefore not included during analyses. However, of the participants reporting recent medication use, very few indicated that they had taken any antihelminth medication in the past month (n = 35, 7% of those reporting medication use). This finding is consistent with a lack of accessible medical care in most communities included in this study. In addition, ~80% of the participants missing data on antihelminth drug use were infected with at least one STH, suggesting most of these participants had not recently taken antihelminth medication. Moreover, of the 35 individuals reporting antihelmintic medication use in the past month, many still contained STH ova in their stool (n = 16), indicating these medications had not yet had an effect or had failed to clear the infection. From the reported cases of medication use, this factor does not appear to be an important determinant of STH infection patterns in this population.

## Conclusions

In summary, the present study examined how aspects of MI shape STH infection patterns among a population characterized by a heavy infectious disease burden. To better test the relationship between MI and infection patterns, this research used a large study sample and assessed how household MI was associated with STH infection status and intensity. Hypothesized links between STH infection patterns and household MI level (as reflected by a composite measure of household infrastructure and key infrastructure elements) were tested. The results indicate that, as expected, participants living in more market integrated households exhibited significantly lower odds of STH infection and lower infection intensity (EPG) values, for both *A*. *lumbricoides* and *T*. *trichiura*. These findings also indicate that floor material and water source may represent particularly important sources of STH infection exposure. Overall, this study identifies key lifestyle factors that should be examined in future efforts to improve health outcomes among groups experiencing the effects of widespread STH infection and rapid MI.

## Supporting information

S1 Checklist STROBE checklist. (DOC)

S1 Table Descriptions of data collection year, number of participants, location, and the general characteristics of each community included in the study sample. (DOCX)
