# Supplementary material for: Market integration and soil-transmitted helminth infection among the Shuar of Amazonian Ecuador
Source: PLoS One. 2020 Jul 31;15(7):e0236924. doi: 10.1371/journal.pone.0236924 (PMC7394393; doi:10.1371/journal.pone.0236924)
Supplement: S1 Table — (DOCX) [file pone.0236924.s002.docx]

**S1 Table. Community descriptions.** Descriptions of data collection year, median household style of life score, number of participants, location, and the general characteristics of each community included in the study sample. Communities are listed in order of median household style of life scores, which serves as an approximation of community market integration level. Community median style of life scores are presented from lowest to highest (i.e., community 1 has the lowest median score, while community 10 has the highest).

| **Community (year data collected; median household style of life score, min -max score)** | **Sample size** | **Location description** | **General level of Market Integration** |
| --- | --- | --- | --- |
| **1**  **(2012; 4, 2-6)** | 63 | Located along the banks of the Morona river in the Amazon jungle, generally hot, humid, and wet | Several houses with electricity, many houses traditionally built, some water pumps, no designated bathrooms |
| **2**  **(2012; 7, 1-11)** | 60 | Located along the banks of the Morona river in the Amazon jungle, generally hot, humid, and wet | No electricity (some solar panels), houses built in traditional style, no wells or bathroom facilities |
| **3**  **(2016; 9, 6-14)** | 44 | ~30-minute walk from the Morona river in the Amazon jungle, generally hot, humid, and wet | Some houses with electricity, fewer houses traditionally built, communal water pump |
| **4**  **(2017; 9, 7-15)** | 10 | Situated at a slightly higher elevation than the jungle communities; hot and humid during the day, but cooler nights | Electricity, most houses built in traditional-style, only one house with a well and designated bathroom |
| **5**  **(2017; 10, 10-18)** | 18 | Situated at a slightly higher elevation than the jungle communities; hot and humid during the day, but cooler nights | Most houses without electricity, no traditional-style houses, many bathrooms with running water and spigots for drinking water |
| **6**  **(2016; 12, 3-16)** | 92 | Situated at a slightly higher elevation than the jungle communities; hot and humid during the day, but cooler nights | Electricity, very few traditional-style houses, many bathrooms with running water and spigots for drinking water |
| **7**  **(2013; 12, 3-18)** | 175 | Located along the banks of the Morona river in the Amazon jungle, generally hot, humid, and wet | Mix of house types, fairly reliable electricity, some houses with wells and designated latrines |
| **8**  **(2011; 13, 7-17)** | 72 | Positioned in the Upano River Valley, slightly cooler and drier than jungle communities | Electricity, many government-built houses, and school bathroom with running water |
| **9**  **(2014; 14, 7-17)** | 70 | Positioned in the Upano River Valley, slightly cooler and drier than jungle communities | Electricity, many government-built houses, all water from a tube or well, some bathrooms with running water |
| **10**  **(2017; 14, 7-19** | 16 | Situated at a slightly higher elevation than the jungle communities; hot and humid during the day, but cooler nights | Electricity, no traditional-style houses, many bathrooms with running water and spigots for drinking water |
